# Partial normalization of microbiota dysbiosis in condyloma acuminatum patients following treatment

**DOI:** 10.3389/fcimb.2025.1558469

**Published:** 2025-03-31

**Authors:** Kai Chen, Meng Li, Tian-Qi Fu, Yan-Yan Hu, Lan Chen, Qian Huang, Li Xu, Zhi-Liang Zeng, Dong-Sheng Li

**Affiliations:** ^1^ Department of Dermatology, Traditional Chinese and Western Medicine Hospital of Wuhan, Tongji Medical College, Huazhong University of Science and Technology, Wuhan, China; ^2^ Research Center for Infectious Skin Diseases of Hubei Province, Wuhan No. 1 Hospital, Wuhan, China; ^3^ Hubei Province & Key Laboratory of Skin Infection and Immunity, Wuhan No. 1 Hospital, Wuhan, China; ^4^ School of Medicine, Jianghan University, Wuhan, China

**Keywords:** condyloma acuminatum (CA), skin microbiome, microbiota dysbiosis, microbial interaction and function, dysbiosis resolution

## Abstract

**Introduction:**

Condyloma acuminatum (CA) is the most common sexually transmitted disease and the presence of microbiota dysbiosis has been observed to promote the progress of the disease. However, the explicit characteristics of microbiota dysbiosis in CA patients have not been well elucidated yet.

**Methods:**

We recruited 40 CA patients who received QYXJ (an in-hospital prescription that has been used to treat CA for many years) treatment and 40 healthy controls (HC) in the current study. Before and after two weeks QYXJ administration, the skin microbiome of each patient was assessed using 16S rRNA gene sequencing.

**Results:**

Here, we found increased relative abundances of *Staphylococcus* and *Lactobacillus*, whereas a decreased *Escherichia* in CA patients relative to healthy controls (HC). Moreover, we also observed significant alpha and beta diversity differences between the CA and HC groups, and QYXJ treatment effectivity attenuated these alterations of genus level and microbial diversity in patients with CA. Importantly, further microbial interaction and function analyses revealed the significantly enriched relative abundance of *Caldivirga* and *Streptococcus* in microbial community, decreased complexity of microbial interactions and downregulated metabolic pathways in CA patients, including membrane transport, lipid metabolism and carbohydrate metabolism. Remarkably, QYXJ administration partially restored these microbiota dysbiosis, which subsequently shifts microbiomes of patients with CA towards healthy-like microbiota.

**Conclusion:**

This study further confirmed the changes of skin microbiome in CA pathogenesis and firstly revealed the protective effects of QYXJ in microbiota dysbiosis resolution, suggesting its potential role as a novel method for CA treatment.

## 1 Introduction

Condyloma acuminatum (CA) is the most common sexually transmitted disease and is mainly caused by human papillomavirus (HPV) infection, predominantly affecting the genital and anal regions (Park et al., 2015; Zhou et al., 2022). The World Health Organization reports that CA is a widespread disease and the global incidence of CA is 160 to 289 per 100,000 people each year, with an increasing trend year by year (Patel et al., 2013). According to the carcinogenic capacity, HPV genotypes can be divided into low-risk (non-cancer-related type) and high -risk (cancer-related type) types (Della Fera et al., 2021). Up to now, more than 200 types of HPV have been found, of which more than 40 types are related to CA (Hua et al., 2021). It was reported that about 90% of CA are caused by low-risk type HPV infection, with HPV 6 and 11 as the most common type, and high-risk type like HPV 16, 18, 30, 31, 35, 42, 43 have also been detected occasionally (Lorincz et al., 2002; Cong et al., 2016; Wang et al., 2019). Once infected with high-risk type, CA patients may develop into penile cancer and cervical cancer (Sakamoto et al., 2019; Piña-Sánchez, 2022). Although low-risk HPV infection don’t induce reproductive tract malignant tumors, CA patients are usually accompanied with other sexually transmitted diseases, which leading to a worse prognosis and recurrence of CA (Zhou et al., 2019). Moreover, HPV virus also has been shown to damage the human immune system and the reproductive organs of CA patients (Sand and Thomsen, 2017). Therefore, HPV infection (especially low-risk type) is a major pathogenic factor to cause the occurrence and recurrence of CA, but the underlying potential mechanisms are yet to be identified.

Vaginal microbiota (VMB) is an important part of vaginal microenvironment that maintains the vaginal health and is the first line of defense against sexually transmitted infections (Kalia et al., 2020). Previous research reported that *Lactobacillus* are the key microbes in healthy women and maintains a low pH vaginal environment to preventing growth of other bacteria (Kwon and Lee, 2022). Moreover, the stability of the cervicovaginal microbial composition was commonly associated with the prevalence of *Lactobacillus* and a low microbial diversity, and another clinical study reported the association between a higher vaginal microbial diversity and the development of squamous intraepithelial lesions (SIL) (Curty et al., 2019; Li et al., 2024). Therefore, when the factors like HPV infection can lead to dysbiotic vaginal microbiota and the physical, chemical, and immune barrier functions of the skin are damaged, which increasing the susceptibility to infection by other microorganisms (Liu et al., 2016; Harris-Tryon and Grice, 2022). In addition, the infection of specific microorganisms also makes the treatment of HPV more difficult and complicated (Karim et al., 2018; Romero-Morelos et al., 2019). Meanwhile, there is also a study reported that HPV infection is associated with the imbalance of vaginal microbiota and the vaginal microbiota is strongly associated with vaginal inflammation and carcinogenesis (Santella et al., 2022). Thus, the microbiome of CA patients will be changed because of HPV infection. However, the explicit characteristics of microbiota dysbiosis in CA patients are yet to be well elucidated, and whether microbiota dysbiosis is the cause or result of CA requires further investigation.

In this study, we analyzed the microbial community compositions, calculated the microbial alpha and beta diversity, and explored the microbial co-occurrence networks and functions in healthy controls and CA patients (before and after received a two weeks QYXJ treatment), to identify the explicit characteristics of microbiota dysbiosis in CA patients and reveal the protective effects of QYXJ in microbiota dysbiosis resolution.

## 2 Methods

### 2.1 Study design

In this study, a total of 40 patients who were diagnosed with CA and received QYXJ (an in-hospital prescription that has been used to treat CA for many years) treatment (dilute warm baths for 20 minutes, twice daily) in the dermatology department of Wuhan No. 1 Hospital, from May 2023 to June 2024 were recruited, and 40 individuals who underwent physical examination were enrolled as the comparable healthy controls (HC). Before and after two weeks treatment, the healthy or lesional tissue were collected as previously described (Li et al., 2021). All the participants have signed the informed consent before the study and the demographic data from all participants were listed in **Table 1**. This study was approved by the ethics committee of Wuhan No. 1 Hospital (2022S042).

**Table 1:** Demographic characteristics of the patients in this study.

Characteristics	HC group (n=40)	CA group (n=20)	*p* value
Age, years (mean ± SEM)	35.78 ± 2.160	29.35 ± 1.800	> 0.05
Male, n (%)	31 (77.5)	16 (80)	> 0.05
Distribution, n (%)			
Perianal	**-**	2 (10.00)	
Genitals	**-**	16 (80.00)	
Perianal and genitals	**-**	1 (5)	
Urethral	**-**	1 (5)	

### 2.2 Sample collection

After treatment, only 20 CA patients returned to the hospital and completed the sample collection. Samples obtained from the skin were collected with a sterile swab and placed in a 5 ml sterile plastic tube, and immediately frozen at -80°C for later DNA extraction.

### 2.3 DNA extraction and 16S rRNA gene sequencing

Microbial DNA was extracted from each sample using an Ezup column DNA extraction kit (Sangon Biotech, Shanghai, China) according to the manufacturer’s instructions. The V4-V5 region of the microbial 16S ribosomal RNA gene was amplified by PCR (94°C for 3 min, followed by 30 cycles at 94°C for 40 s, 56°C for 60 s, and 72°C for 60 s and a final extension at 72°C for 10 min) using primers 515F (5’- GTGCCAGCMGCCGCGG-3’) and 909R (5’-CCCCGYCAATTCMTTTRAGT-3’). Purified amplicons (concentration >10 ng/μl) then were sequenced (2X250 bp pairwise) on a MiSeq platform (Illumina, San Diego, United States). The original 16S rRNA FASTQ sequences were uploaded to a public database on the European Nucleotide Archive (ENA, http://www.ebi.ac.uk/ena) (accession number: PRJEB80314).

### 2.4 Bioinformatic analysis

The raw FASTQ sequencing data were performed sequence stitching and quality trimming by the QIIME (quantitative insights into microbial ecology) pipeline (version 1.7.0) (Zhu et al., 2022). Chimeric sequences were identified and removed using Usearch (version 7.0) and the remaining sequences were clustered to generate operational taxonomic units (OTUs) with a 97% similarity identity using UPARSE (http://drive5.com/uparse/) (Edgar, 2013). Then the OTUs were annotated with taxonomic information according to a ribosomal database project (RDP) database (Cole et al., 2014). In addition, alpha diversity (Observed species and Shannon’s index) and beta diversity (based on the Bray-Curtis and Jaccard distance matrices) of microbial communities were visualized by principal co-ordinates analysis (PCoA), and predictive analyses of microbial functions were performed using a PICRUSt2 (phylogenetic investigation of communities by reconstruction of unobserved states) software (Douglas et al., 2020).

### 2.5 Statistical analysis

The differences in microbial relative abundance between groups were calculated by the analysis of variance (ANOVA). The differences in alpha diversity were calculated by the Kruskal-Wallis (K-W) test and the differences in beta diversity were calculated by the permutational multivariate analysis of variance (PERMANOVA). Spearman’s correlation was calculated in R using Hmisc package and the correlation with *p* < 0.05 and |r| > 0.4 was selected for subsequent analysis. Microbial co-occurrence network analyses were performed using Gephi software (version 0.10.1) and the functional differences were analyzed using STAMP (statistical analysis of metagenomic profiles) software. *P* < 0.05 was considered to be statistically significant in this study.

## 3 Results

### 3.1 Microbial community composition and diversity

**Figure 1:**
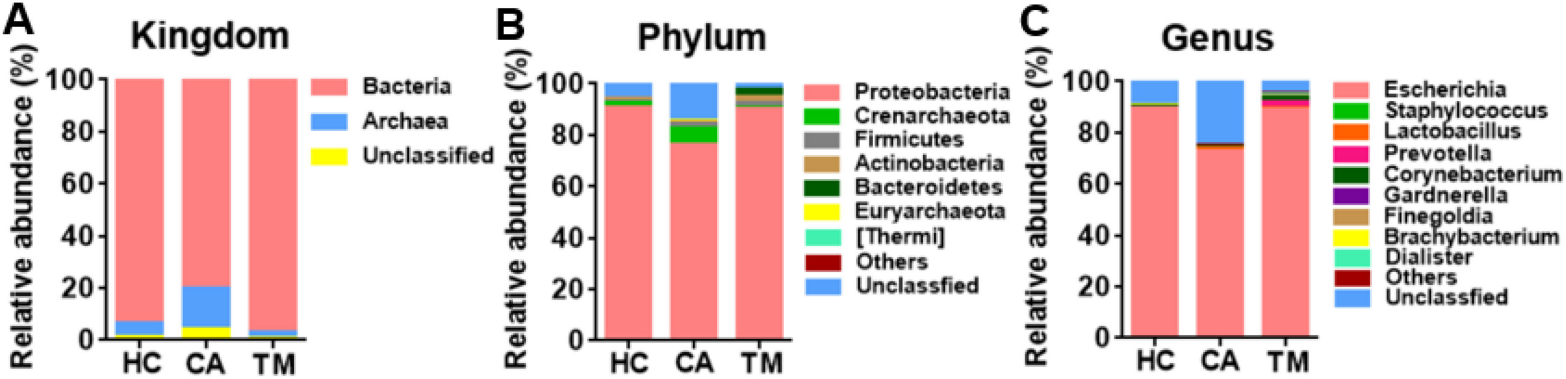
Microbial community composition in different groups. **(A)** Relative abundance of skin microbiota at the kingdom level in the three groups. **(B)** Relative abundance of skin microbiota at the phylum level in the three groups. **(C)** Relative abundance of skin microbiota at the genus level in the three groups. HC, healthy control group; CA, CA patient group, before treatment; TM, CA patient group, after two weeks treatment.

To explore how the skin microbiome changed among groups, relative abundance at kingdom, phylum and genus levels was analyzed. Bacteria were the dominated microbe among three groups (**Figure 1A**), while *Proteobacteria*, *Crenarchaeota* and *Firmicutes* dominated the microbial community at the phylum level (**Figure 1B**). Additionally, *Escherichia*, *Staphylococcus* and *Lactobacillus* were the most abundant genera and the alterations of these three microbes were observed among groups. Compared to the healthy controls (HC) group, decreased *Escherichia* (72.47 *vs*. 89.27%, *p* < 0.05), increased *Staphylococcus* (0.25 *vs*. 0.09%, *p* < 0.05) and *Lactobacillus* (1.30 *vs*. 0.08%, *p* < 0.05) were detected in the CA group. However, QYXJ treatment attenuated the changes in these genera (*Escherichia*, 88.92 *vs*. 72.47%, *p* < 0.05; *Staphylococcus*, 0.13 *vs*. 0.25%, *p* < 0.05; *Lactobacillus* 0.31 *vs*. 1.30%, *p* < 0.05) (**Figure 1C**).

**Figure 2:**
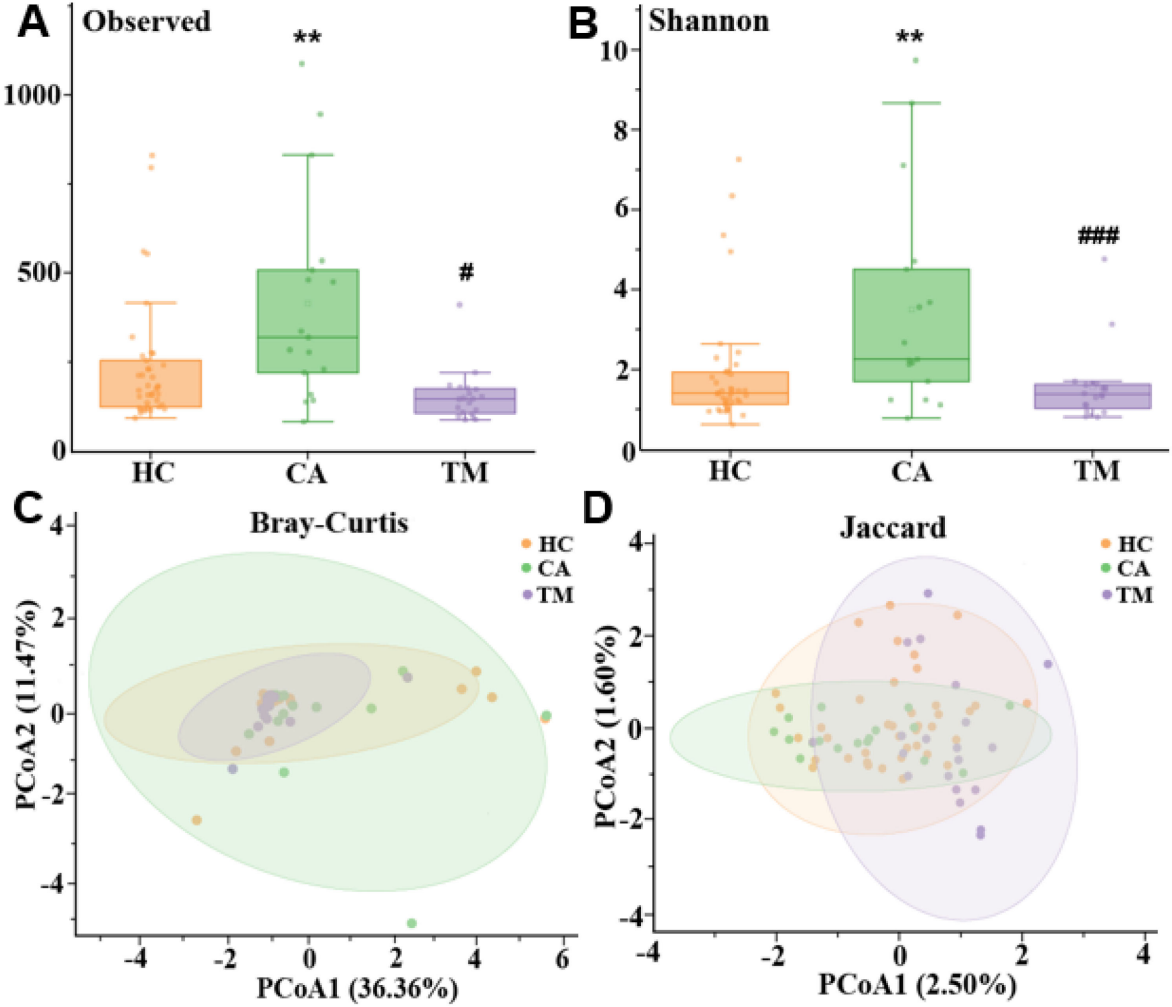
Alpha and beta diversity in different groups. **(A, B)** Analysis of alpha diversity of skin microbiota in different groups by Observed analysis **(A)** and Shannon analysis **(B)**. *, CA *vs*. HC; #, TM *vs*. CA, Kruskal-Wallis test. **p < 0.01, #*p* < 0.05, ###*p* < 0.001. **(C, D)** Principal coordinate analysis (PCoA) plots of beta diversity based on Bray-Curtis analysis **(C)** and Jaccard analysis **(D)** in different groups. HC, healthy control group; CA, CA patient group, before treatment; TM, CA patient group, after two weeks treatment. *P*-value was estimated by permutational multivariate analysis of variance (PERMANOVA).

In order to distinguish the species diversity of each group, we calculated the alpha diversity of skin microbiota in different groups by Observed analysis and Shannon analysis, respectively. Comparing with the HC group, we found that the alpha diversity was significantly increased in the CA group, while QYXJ treatment markedly decreased the alpha diversity of the CA patients, as revealed by the Observed and Shannon diversity indices (**Figures 2A, B**). We then analyzed the differences of microbial communities between groups. Principal coordinate analysis (PCoA) shown that the plots in the HC group and the TM group are clustered together whereas the plots in the CA cluster are dispersed (**Figure 2C**). Moreover, permutational multivariate analysis of variance (PERMANOVA) revealed significant differences in microbial beta diversity between CA and HC groups (Bray-Curtis, *p*=0.026; Jaccard, *p*=0.011) (**Figures 2C, D**). Remarkably, QYXJ treatment also significantly alleviated the beta diversity of the CA group (Bray-Curtis, *p*=0.003; Jaccard, *p*=0.001) (**Table 2**). Taken together, these results revealed the changes of microbial community composition and diversity in patients with CA, and QYXJ treatment effectively alleviates the microbiota dysbiosis of the CA patients.

**Table 2:** Permutational multivariate analysis of variance (PERMANOVA) revealed significant differences in beta diversity between groups.

PERMANOVA	Bray-Curtis			Jaccard		
	F	R2	p	F	R2	p
CA vs HC	5.386	0.092	**0.026**	4.561	0.078	**0.011**
TM vs CA	7.807	0.191	**0.003**	7.751	0.181	**0.001**

Bold values indicated the p<0.05.

### 3.2 Significantly different operational taxonomic units and microbial co-occurrence network

**Figure 3:**
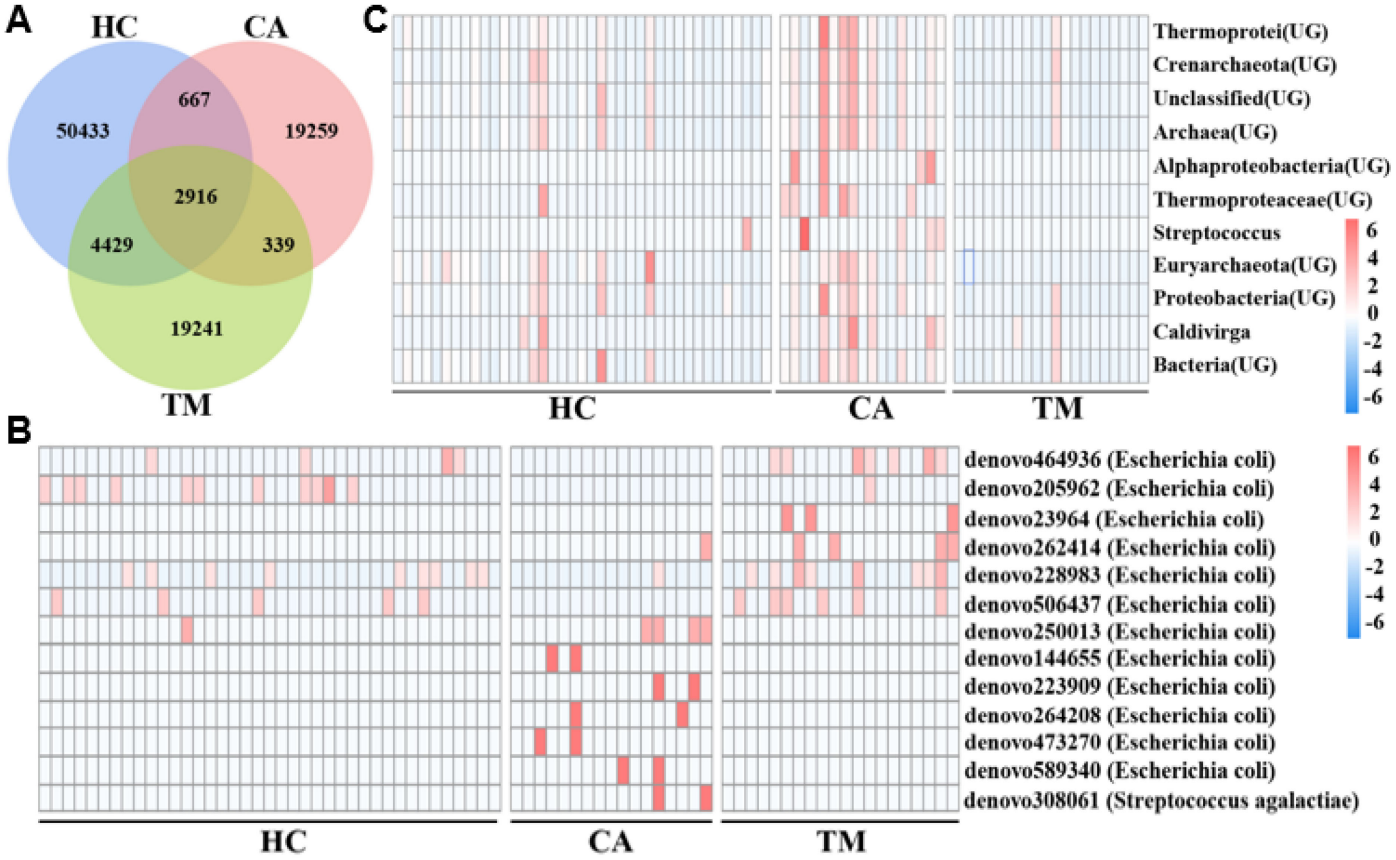
Enrichment analysis of microbial operational taxonomic units (OTUs) and communities in different groups. **(A)** Venn diagram displaying the distribution of the common and unique OTUs in the three groups. **(B)** Heatmap plot showing the representatively different OTUs in each group. **(C)** Heatmap plot showing the most abundant microbial communities in each group. HC, healthy control group; CA, CA patient group, before treatment; TM, CA patient group, after two weeks treatment. Significant difference was estimated by analysis of variance (ANOVA, *p* < 0.05).

To better understand the microbial diversity among the three groups, we constructed Venn diagrams to illustrate the common and unique OTUs of each group. As shown in **Figure 3A**, a total of 50433 OTUs were uniquely identified in the HC cluster, 19259 OTUs were uniquely identified in the CA patients, 19241 OTUs were uniquely identified in the TM group, and 2916 OTUs were commonly identified in the three groups. By comparing the OTUs between groups, we found that the HC group has the fewer common OTUs with the CA group (667), whereas has the more common OTUs with the TM group (4429). These results suggested that QYXJ treatment partially rescue the skin microbiota dysbiosis of the CA patients. According to the abundance of OTU in each sample, enrichment analysis of significantly different OTUs in the three groups were displayed in a heatmap plot (**Figure 3B**). In comparison with the HC group, the relative abundance of some microbiomes at the phylum level of *Proteobacteria* (*Thermoprotei, Caldivirga*) and *Firmicutes* (*Bacilli*, *Streptococcus*) were significantly enriched in the CA patients (*p* < 0.05). However, QYXJ treatment obviously decreased the abundance of *Caldivirga* and *Streptococcus* in the TM group (*p* < 0.05) (**Figure 3C**).

**Figure 4:**
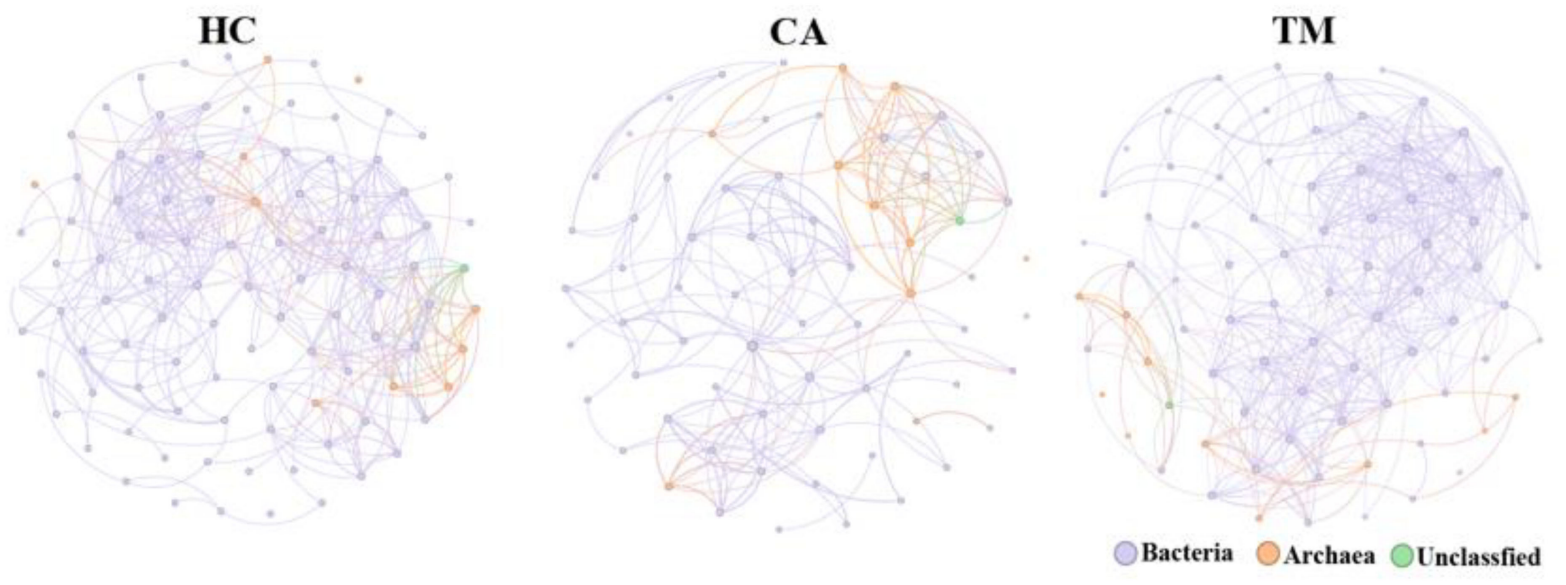
Correlation analysis reveals the microbial interactions in each group. Spearman’s rank correlation test, *p* < 0.05, |r| > 0.4. HC, healthy control group; CA, CA patient group, before treatment; TM, CA patient group, after two weeks treatment.

Co-occurrence networks inferred from the abundance data of microbial communities have been widely developed to predict microbial interactions that shape the structure and function of microbial communities (Riera and Baldo, 2020). We then performed co-occurrence network analysis to explore the microbial interactions in each group. After removing unconnected nodes, the final network consists of 99 nodes and 404 edges (387 co-occurrence edges) in HC group, 68 nodes and 191 edges (180 co-occurrence edges) in CA patients, 80 nodes and 456 edges (439 co-occurrence edges) in TM group (**Figure 4**, **Table 3**). Our results suggesting a co-occurrence pattern in these microbial networks. Moreover, we also observed that the nodes, edges and average degree in CA group are small in comparison with the co-occurrence network in HC group. However, QYXJ treatment increased the clustering coefficient and decreased the modularity of the co-occurrence network in CA patients (**Figure 4**). Collectively, these results demonstrated that QYXJ treatment remarkably decreases the relative abundance of *Caldivirga* and *Streptococcus* in microbial community, and increases the complexity of microbial interactions in CA patients.

**Table 3:** Topological features of microbial co-occurrence network in different groups.

Characteristic	HC	CA	TM
Nodes	99	68	80
Edges	404	191	456
Average degree	8.162	5.618	11.4
Average path length	3.357	3.472	2.747
Graph diameter	9	9	7
Graph density	0.083	0.084	0.144
Clustering coefficient	0.545	0.659	0.666
Modularity	0.546	0.626	0.443
P (%)	95.79	94.24	96.27

### 3.3 Functional analysis reveals obviously different metabolic pathways among groups

**Figure 5:**
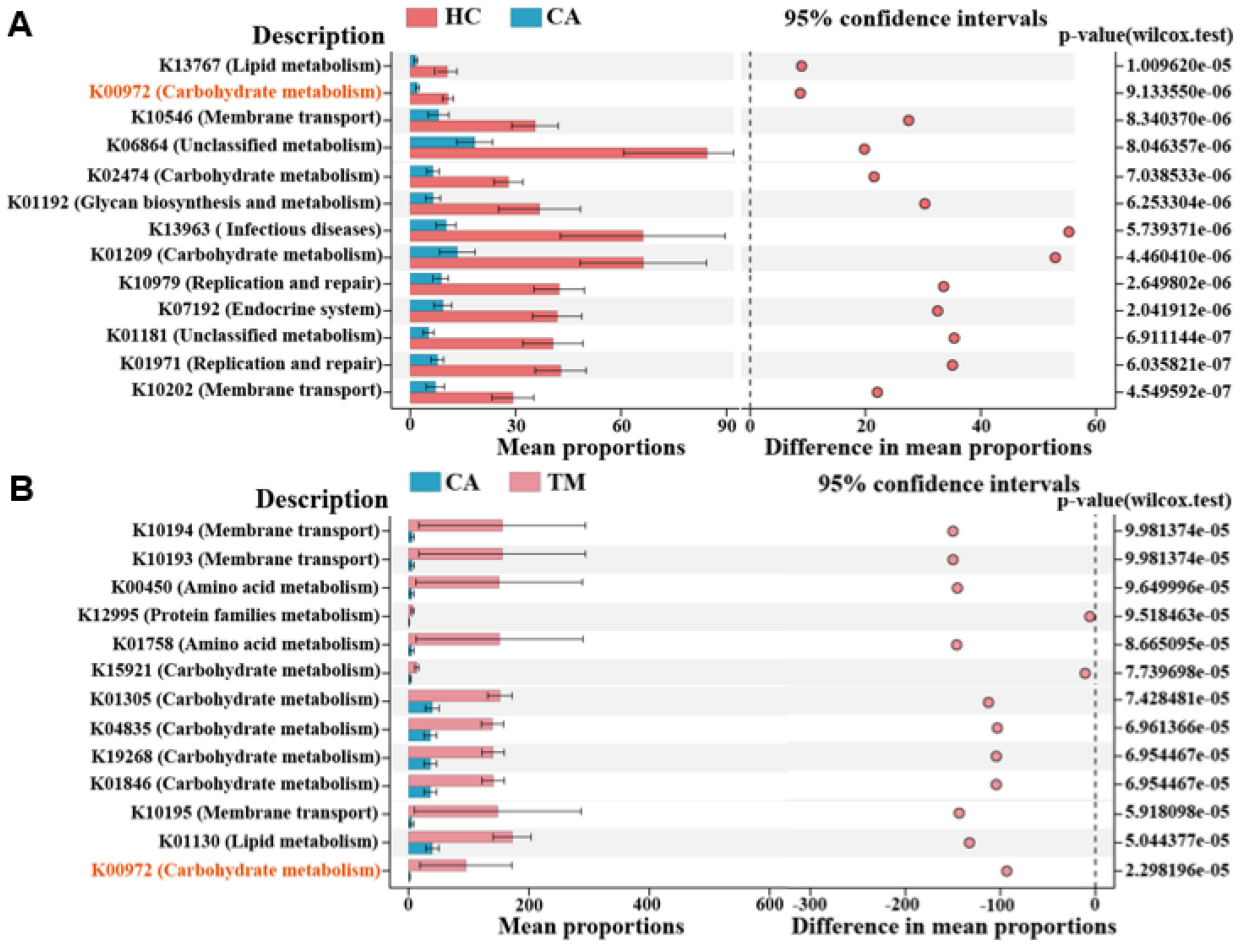
Functional analysis reveals obviously different metabolic pathways in different groups. **(A, B)** The significantly altered microbial functional pathways in CA *vs*. HC **(A)** and TM *vs*. CA **(B)**, as revealed by the extended error bar method. Wilcoxon rank sum test. HC, healthy control group; CA, CA patient group, before treatment; TM, CA patient group, after two weeks treatment.

To further investigate the differential function of the microbial communities among groups, we finally performed phylogenetic investigation of communities by reconstruction of unobserved states (PICRUSt) to predict microbial functional pathways (Hu et al., 2022). As shown in **Figure 5A**, 13 significantly enriched KEGG pathways were selected between CA and HC groups. We found that several metabolic pathways are downregulated in the CA group in comparison with the HC group, including membrane transport, lipid metabolism and carbohydrate metabolism. Expectedly, QYXJ treatment visibly enhanced the enrichment of these metabolic pathways in the CA group (**Figure 5B**). Importantly, a carbohydrate metabolism pathway (K00972) was significantly downregulated in CA patients and markedly reversed after QYXJ administration (**Figure 5**). Overall, these findings strongly suggested the metabolic dysregulation of skin microbiome in CA patients and QYXJ alleviated microbiota dysbiosis of CA patients through targeting these metabolic pathways.

## 4 Discussion

In the current study, we reported a more accurate and comprehensive understanding of microbiota dysbiosis, including the alterations of *Staphylococcus*, *Lactobacillus* and *Escherichia* in CA patients. As reported, HPV infection caused vaginal microbiota changes in Chinese women and *Lactobacillus* derivatives served as sensors for changes in the vaginal microenvironment (Chee et al., 2020; Zhang et al., 2022). Another research found that *Staphylococcus aureus* is an opportunistic pathogen which can lead to vaginal dysbiosis, aerobic vaginitis, or life-threatening disorders including aerobic vaginitis (AV) and menstrual toxic shock syndrome (mTSS) (Maduta et al., 2024). Similarly, previous studies reported that penile microbiome shape the local micro-environment and are associated with local inflammation and HIV susceptibility, and modify the penile microbiome could potentially prevent HIV transmission (Liu et al., 2017; Prodger et al., 2021; Mehta et al., 2022). Moreover, 16S rRNA gene sequencing revealed that penile microbiome is involved in penile squamous cell carcinoma and the greater relative abundances of *Staphylococcus* in penile microbiome of HIV-infected men also are observed (Onywera et al., 2020; De Deus et al., 2024). Thus, our results will help to clarify the relationship between microbiota dysbiosis and the occurrence of CA.

Importantly, we also confirmed the previous findings of increased alpha diversity in CA patients relative to healthy controls (HC) and a significant difference in microbial beta diversity between the CA and HC groups (Zhou et al., 2019). It is known that the diversity of vaginal microbiota as a modifier of HPV infection and the alpha diversity was significantly higher in HPV-infected group than in healthy control group (Chao et al., 2019; Zhang et al., 2022). Notably, cervical intraepithelial neoplasia progression is associated with increased vaginal microbiome diversity (Chen et al., 2020), and the highest microbial alpha diversity was found in multiple high-risk HPV infected women compared with other infections (Liu et al., 2022). These findings suggesting the interactions between microbial diversity and the risk of HPV infection or CA progression. Moreover, we also demonstrated that QYXJ treatment obviously decreases the alpha diversity of skin microbiome and remarkably attenuates the difference in species composition. Overall, these results confirmed the changes of skin microbiome in CA patients and revealed the association between microbial diversity and CA progression.

Simultaneously, a previous study reported the existence of cervical microbial diversity and compositional differences between patients with cervical dysplasia and cervical cancer, and demonstrated that the *Proteobacteria* was particularly enriched in cervical cancer patients (Sims et al., 2020). Another recent research using meta-analysis revealed the microbial dysbiosis and reported similar results that the opportunistic pernicious microbes *Streptococcus* was enriched in cervical carcinogenesis (Li et al., 2024). Consistent with these reports, we found that the relative abundance of *Proteobacteria* (especially *Caldivirga*) and *Firmicutes* (especially *Streptococcus*) were significantly enriched in CA patients. These results suggested that the enriched *Caldivirga* and *Streptococcus* are associated with the development and progression of CA. In agreement with this, Gen Wei and colleagues demonstrated that a probiotic nanozyme hydrogel regulates vaginal microbiota via decreasing the proportion of *Proteobacteria* to reduce the recurrence of candida vaginitis (Wei et al., 2023). Remarkably, we also observed that QYXJ treatment resulted in a lower proportion of *Caldivirga* and *Streptococcus* to reshape a healthy microbiota in CA patients. Collectively, we demonstrated the microbial composition changes in CA patients, but the direct role of *Caldivirga* or *Streptococcus* alterations in the disease progression of CA needs to be further investigated.

Perturbations of the microbial community composition accompany alterations in microbial metabolites that damage the immune barrier, increase the susceptibility to infection and promote the disease progression (Chen et al., 2018; Dong et al., 2023). However, little is known about the microbial metabolite changes with the skin microbiota dysbiosis in CA patients. Here, we reported that several metabolic pathways, including membrane transport, lipid metabolism and carbohydrate metabolism are downregulated in CA patients in comparison with the healthy controls. Moreover, we also found that QYXJ treatment partially restored the enrichment of these metabolic pathways in patients with CA, which subsequently shifts microbiomes towards healthy-like microbiota. Consistent with these findings, previous studies reported that vaginal microbiota transfer (VMT) partially normalizes the microbiome and neurodevelopment of cesarean-born infants via regulating the metabolic functions of amino acid and carbohydrate metabolisms (Dominguez-Bello et al., 2016; Zhou et al., 2023). Another similar study also found a successful VMT with dysbiosis resolution and live birth after recurrent pregnancy loss (Wrønding et al., 2023). Taken together, these results suggested that dysbiosis resolution is a potential target for various microbial dysbiotic diseases and QYXJ is an effective therapeutic approach for these diseases. Because of the relatively small sample size and the single center in this study, collected more samples in multiple centers are needed. Moreover, further research on the role of microbial metabolite changes in the development of CA and the clinical application of QYXJ in treatment of other dysbiotic microbiota-related diseases are required.

## Data Availability

The datasets presented in this study can be found in online repositories. The names of the repository/repositories and accession number(s) can be found in the article/supplementary material.
